# Impact of skin pigmentation on photoacoustic tomography: a phantom study

**DOI:** 10.1117/1.BIOS.3.2.022510

**Published:** 2026-06-03

**Authors:** Rianne F. G. Bulthuis, Nan S. Lubbers, Felix Lucka, Wiendelt Steenbergen, Nienke Bosschaart, Srirang Manohar

**Affiliations:** a University of Twente, Department of Biomedical Imaging and Sensing, Multi-Modality Medical Imaging, Enschede, The Netherlands; b Centrum Wiskunde and Informatica, Amsterdam, The Netherlands; c University of Twente, Department of Biomedical Imaging and Sensing, Biomedical Photonic Imaging, Enschede, The Netherlands

**Keywords:** skin color, skin pigmentation, photoacoustic tomography, phantom, breast imaging.

## Abstract

**Significance:**

Skin pigmentation is a known source of bias in optical and photoacoustic imaging. Although these effects are well investigated for two-dimensional photoacoustic imaging systems, their impact on three-dimensional (3D) photoacoustic tomography remains largely unexplored.

**Aim:**

We aim to generate insights into the impact of skin pigmentation on the performance of 3D breast photoacoustic tomography using phantoms.

**Approach:**

We developed hemispherical breast phantoms compatible with a multi-wavelength photoacoustic–ultrasound tomography system. The phantoms incorporate a thin skin-mimicking layer over an adipose-mimicking bulk material. Four phantoms with identical geometry and increasing pigmentation levels were fabricated. Embedded wall-less channels filled with sulfate solutions of varying concentrations enabled depth- and spectral-dependent analysis.

**Results:**

Increasing pigmentation led to stronger skin photoacoustic signals, reduced signal-to-background ratios at depth, and increased reflection-related background artifacts. Spectral measurements revealed pigmentation-dependent attenuation and reduced chromophore contrast in the wavelength range relevant for oxygen saturation estimation.

**Conclusions:**

Three-dimensional photoacoustic tomography is susceptible to pigmentation-induced bias affecting imaging depth and spectral analysis. The presented phantom provides a validated platform for developing strategies to improve the reliability and inclusivity of photoacoustic tomography imaging across skin tones.

Statement of Discovery (1) This work shows for the first time the impact of skin color in 3D photoacoustic tomography. (2) This work provides a validated phantom platform for developing strategies to improve the reliability and inclusivity of 3D photoacoustic tomography imaging across skin tones.

## 1 Introduction

Skin pigmentation can introduce significant biases in many optical techniques, because the availability of light fluence inside tissue is strongly influenced by optical absorption of melanin in the skin.^1^ These biases have been reported in transcutaneous bilirubinometers,^2^^,^^3^ where optical absorption by melanin interferes with the spectral algorithms used to estimate bilirubin levels, leading to reduced accuracy in individuals with darker skin. Similar pigmentation-related biases have also been reported in techniques such as near-infrared spectroscopy^4^ and pulse oximetry. Problems with the latter gained wide attention in the context of COVID-19, when a study showed that pulse oximetry demonstrated reduced accuracy in darker skin.^5^

Similar concerns have been identified in photoacoustic (PA) imaging. This hybrid modality provides optical contrast via absorption of light by tissue chromophores, and the detection of the resulting ultrasound waves enables high spatial resolution.^6^ Because PA imaging relies on optical absorption as its source of contrast, high pigmentation levels result in strong photoacoustic signals from the skin and limit light penetration into deeper tissues. Several studies reported that this leads to reduced imaging depth, artifacts that obscure vascular features, and systematic underestimation of blood oxygen saturation.^7^^–^^12^

Investigations into the effect of skin pigmentation on PA imaging have thus far been limited to two-dimensional (2D) imaging systems. These systems use linear or curved ultrasound detection arrays in contrast to three-dimensional (3D) PA tomography systems. In the latter, not only detection but also the illumination geometry is arranged around the object, enabling 3D image reconstruction.^13^ The impact of light absorption in highly pigmented skin on PA tomography imaging is yet to be determined.

The effect of skin pigmentation on PA imaging has been thoroughly studied in phantoms, which provide a controlled and reproducible platform.^14^^,^^15^ Phantoms containing a skin-mimicking layer have been developed for 2D PA imaging systems,^16^^,^^17^ but their planar geometry is incompatible with 3D PA tomography, which requires detection from all sides of the phantom. To date, no suitable phantom exists for PA tomography that incorporates a realistic skin-mimicking layer. Developing such a phantom would enable systematic evaluation of pigmentation-related effects in PA tomography imaging.

The goal of our work is to generate insights into the impact of skin pigmentation on the performance of 3D breast PA tomography. We designed and fabricated skin-mimicking breast phantoms for PA tomography, specifically for the multi-wavelength hybrid photoacoustic–ultrasound breast imaging system (PAM3).^18^ The phantoms are made using a two-stage fabrication process: (i) a skin-mimicking layer, composed of a copolymer-in-oil (CiO) material containing nigrosine as a melanin-mimicking additive, (ii) followed by a bulk material made of native gel wax to mimic adipose breast tissue. Four channels are embedded within the phantom to allow the incorporation of dyes, for which we used sulfate solutions to represent multiple chromophore concentrations.

The phantom design enables controlled variation of pigmentation levels and provides a platform for assessing the confounding influence of skin color on PA tomography. Four phantoms with identical geometry and material composition were fabricated, differing only in pigmentation level of the skin-mimicking layer, ranging from non-pigmented to dark skin tones. The phantoms are characterized in terms of their optical and acoustic properties, validated against the design requirements, and used to demonstrate PA imaging performance using the PAM3 system.

## 2 Methods

### 2.1 Photoacoustic Tomography System

The measurements were conducted using the in-house-built multi-wavelength hybrid PAM3 system.^18^ The system features a water-filled imaging bowl providing hemispherical illumination and detection. During image acquisition, the bowl rotates around the breast. The illumination is provided by forty laser bundle outputs, distributed on the inner surface of the imaging bowl and connected to a tunable laser source covering the range of 680 to 1060 nm. A total of 512 ultrasound transducers are distributed on the inner surface of the imaging bowl. These transducers operate in dual mode, enabling both the detection and emission of ultrasound signals, with a center frequency of 1 MHz (123% bandwidth). A comprehensive description of the PAM3 system and its reconstruction algorithms is provided in Dantuma et al.^18^

### 2.2 Phantom Design

**Fig. 1:**
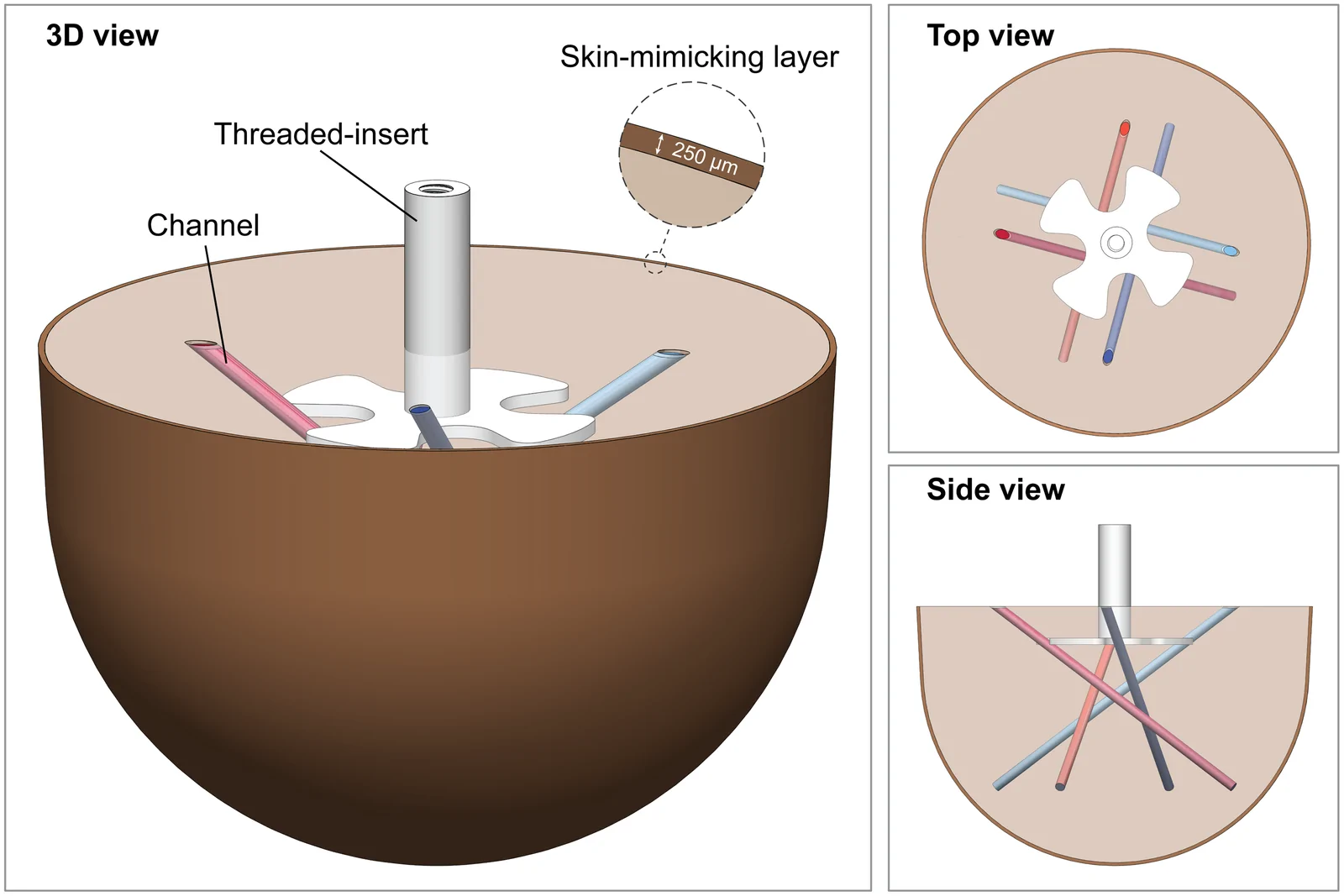
Schematic overview of the breast-mimicking phantom designed for the PAM3 imaging system. The phantom consists of a 6-cm radius hemispherical section on a 3-cm high cylindrical base, includes a 250-μm skin-mimicking layer, and uses adipose-mimicking bulk material. Four 3-mm wall-less channels are embedded to accommodate blood-mimicking fluids with varying oxygen saturation levels.

The phantom was designed for use with the PAM3 system and comprises a hemispherical section with a 6-cm radius attached to a cylindrical base with a 3-cm height (see Fig. 1). This geometry ensures homogeneous optical illumination across the phantom surface. A cast-in threaded insert was embedded in the cylindrical base to position the phantom in the center of the imaging bowl.

The phantom includes a layer that mimics the epidermis. The epidermis contains melanin, which is the primary contributor to skin optical absorption, whereas the other layers do not contain melanin.^19^ To specifically study the effect of skin color, our investigation was limited to the epidermis. Incorporating additional dermal features would increase physiological realism, but this was considered beyond the scope of the present study. The epidermal thickness of the female breast is reported as 76.9±26.2  μm.^20^ However, preliminary tests using various gel formulations, including the material ultimately selected for the phantom (see Sec. 2.3), showed that achieving this thickness for the skin-mimicking layer was not reliably feasible, especially given the curved hemispherical geometry of the phantom. After several fabrication experiments, we were able to achieve a reliable layer thickness of 250  μm. As this study focuses on relative pigmentation effects rather than absolute pigmentation measurements, the increased layer thickness is not likely to affect the conclusions. Similar considerations have been reported before^16^ in a simpler cylindrical phantom where a substantially thicker layer (1 mm) was used due to similar fabrication limitations. Moreover, the PAM3 imaging system has a best spatial of 400  μm, meaning that the skin-mimicking layer thickness remains below this limit, similar to the epidermal layer of the skin. The bulk material of the phantom was intentionally designed to have low optical absorption to enable visualization of the channels. Ideally, it should mimic the optical properties of adipose tissue, which is the primary breast component in the post-menopausal screening population.^21^ However, matching optical absorption at specific wavelengths was not prioritized, as it would require the use of dyes that could introduce additional variability among phantoms. Instead, emphasis was placed on matching the acoustic properties of adipose tissue.

To evaluate the effect of pigmentation on the spectral behavior of chromophores of interest, such as blood, four wall-less channels were created in the bulk (see Fig. 1). They could be filled with solutions with different optical absorption. All channels have a diameter of 3 mm, which is similar to the diameter of large breast vessels.^22^ The channels were equally spaced along the depth of the phantom, with sufficient separation to prevent optical and acoustic shadowing or other interference among them. Although this configuration does not replicate the complexity of clinical vasculature, it allows systematic assessment of depth-dependent signal decay and evaluation of how skin pigmentation affects the spectral response.

### 2.3 Phantom Materials

#### 2.3.1 Skin-mimicking material

Copolymer-in-oil was selected as the base material for the skin-mimicking material due to its tunable optical and acoustic properties.^23^ To reproduce the optical absorption of melanin, nigrosine was incorporated into the base material. Nigrosine was previously identified by Vogt et al.^17^ as an adequate substitute for melanin in poly(vinyl chloride) plastisol. Their method for incorporating nigrosine was adapted for CiO, enabling similar absorption characteristics within a different base material.

The speed of sound of CiO can be adjusted to values within the soft-tissue range (1450 to 1516 m/s) by varying the ratio of polymers.^24^ A combination of 8% low-density polyethylene and 15% styrene-ethylene-butylene-styrene produced the highest speed of sound while allowing thin-layer fabrication.

Four skin-mimicking materials were prepared, representing non-pigmented, light, intermediate, and dark pigmented skin. Pigmentation levels were defined by the melanosome volume fractions ($M_{f}$), with target values of 0%, 1.3 to 6.3%, 11 to 16%, and 18 to 43%, respectively.^25^ The conversion of the $M_{f}$ values into absorption coefficients ($\mu_{a}$), was performed using the approach described in Ref. 26 for the wavelength range 400 to 1100 nm, as given by the following equation:

$$\mu_{a,\text{epidermis}}(\lambda )=(M_{f}\mu_{a,\mathrm{mel}}(\lambda )+(1-M_{f})\mu_{a,0}(\lambda ))(1-C_{H_{2}O})+C_{H_{2}O}\mu_{a,H_{2}O}(\lambda ).$$ (1)

Here, $\mu_{a,\mathrm{mel}}$ is the absorption coefficient of a typical cutaneous melanosome, $\mu_{a,0}$ is the baseline absorption coefficient of epidermal tissue without melanin, $C_{H_{2}O}$ is the volume fraction of water, and $\mu_{a,H_{2}O}$ is the absorption coefficient of water. The $\mu_{a,\mathrm{mel}}$ was calculated using the empirical expression from Jacques^13^

$$\mu_{a,\mathrm{mel}}=(519\;{\mathrm{cm}}^{-1}){\left(\frac{\lambda }{500\;\mathrm{nm}}\right)}^{-3.53}.$$ (2)

Here, λ is the wavelength in nanometers. The $\mu_{a,0}$ was calculated using the empirical non-melanin, non-blood baseline absorption model^25^^,^^27^

$$\mu_{a,0}=7.84\times 10^{7}\lambda^{-3.255}.$$ (3)

No additives were used to modify the reduced scattering coefficient ($\mu_{s}^{\prime }$) across skin tones. This was done intentionally to isolate the effect of absorption (melanin) on photoacoustic imaging performance and to avoid introducing additional variability during fabrication. Although $\mu_{s}^{\prime }$ varies between light and dark skins, intra-group variability is large, and light transmission beyond 0.1 mm can be considered similar across all skin tones.^28^ As our imaging targets lie deeper within the phantom, the impact of scattering variations on observed trends is expected to be minimal.

The absorption coefficient for each pigmentation level was reduced across the full wavelength range to account for the thicker skin-mimicking layer (250  μm). This adjustment ensures that the resulting absorbance ($\mu_{a,\text{epidermis}}\cdot d$, where d denotes the layer thickness) matches *in vivo* values at each wavelength. To achieve the targeted pigmentation levels for the non-pigmented, light, medium, and dark skin-mimicking materials, the materials were prepared using the following fabrication procedure. Alcohol-soluble nigrosine (Sigma-Aldrich, 11099-03-9, St. Louis, Missouri, United States) was dissolved in 95% ethanol (10% v/v of the mineral oil) at concentrations of 0%, 0.006%, 0.021%, and 0.047% w/v, respectively. The resulting solutions were sonicated for 20 min at room temperature to ensure complete dissolution. Mineral oil (Sigma-Aldrich, 8042-47-5) was preheated to 150°C in an oil bath, after which the ethanol–nigrosine solution was added slowly under stirring conditions for at least 15 min. Subsequently, 15% w/w styrene-ethylene-butylene-styrene (Sigma-Aldrich, 66070-58-4) and 8% w/w low-density polyethylene (Sigma-Aldrich, 9002-88-4) were dissolved in the mineral oil mixture. The solution was stirred for 1 h at 150°C to achieve homogeneity and then degassed in a vacuum oven at 150°C to remove air bubbles.

#### 2.3.2 Adipose-tissue-mimicking material

Native gel wax (NGW) was selected as the base material to mimic adipose tissue, due to its tunable optical and acoustic properties.^29^ NGW inherently mimics the optical absorption spectrum of adipose tissue.^21^^,^^29^ The reduced scattering coefficient was adjusted using titanium dioxide (${\mathrm{TiO}}_{2}$) to simulate scattering in adipose tissue.^21^^,^^29^

The speed of sound of NGW (1449  m/s) is within the adipose tissue range (1430 to 1480  m/s).^30^^–^^32^ Acoustic attenuation was adjusted by incorporating paraffin wax to replicate the acoustic attenuation of adipose tissue.^29^^–^^31^

The adipose-tissue-mimicking material was prepared based on the protocol described by Maneas et al.^29^ First, 0.15% w/v ${\mathrm{TiO}}_{2}$ was dispersed in ethanol 99%, corresponding to 2% v/v of the NGW (FF1 003, Mindsets, Saffron Walden, United Kingdom). The solution was sonicated for 1 h at room temperature to ensure homogeneity. NGW was melted in an oil bath at 120°C, after which the ${\mathrm{TiO}}_{2}$–ethanol solution was added slowly under stirring conditions for at least 1 h. Subsequently, 8% w/w paraffin wax (Thermo Fisher Scientific, 8002-74-2, Waltham, Massachusetts, United States) was added and stirred until the mixture was homogeneous. Finally, the solution was degassed in a vacuum oven at 120°C to remove air bubbles.

#### 2.3.3 Sulfate solutions

A mixture of copper sulfate (${\mathrm{CuSO}}_{4}$) and nickel sulfate (${\mathrm{NiSO}}_{4}$) solutions was used to fabricate solutions with clearly distinguishable optical absorption spectra,^33^^,^^34^ allowing evaluation of the spectral coloring effect associated with skin pigmentation.^35^ The solution was water-based and contained in the wall-less phantom channels. Diffusion into the surrounding native gel wax adipose-mimicking material is prevented due to the immiscibility between the water-based solution and the oil-based phantom material. This configuration also provides an acoustic impedance contrast comparable to that between blood and adipose tissue.

The sulfate solutions were prepared to create an isosbestic point with a target value that mimic a total hemoglobin concentration of 150  g/L at 797 nm. Copper sulfate (Sigma-Aldrich, 7758-99-8) and nickel sulfate (Sigma-Aldrich, 10101-97-0) powders were dissolved in Milli-Q water. The target molarities of the four sulfate solutions were determined from their optical absorption coefficient at 797 nm.^36^ The resulting mixtures were as follows: 0.094 M ${\mathrm{CuSO}}_{4}$ with 1.048 M ${\mathrm{NiSO}}_{4}$ (1:1 ratio) (s1), 0.119 M ${\mathrm{CuSO}}_{4}$ with 0.699 M ${\mathrm{NiSO}}_{4}$ (1:1 ratio) (s2), 0.145 M ${\mathrm{CuSO}}_{4}$ with 0.349 M ${\mathrm{NiSO}}_{4}$ (1:1 ratio) (s3), and 0.170 M ${\mathrm{CuSO}}_{4}$ (s4).

### 2.4 Material Characterization

The optical properties of the skin- and adipose-tissue-mimicking materials, including the absorption and reduced scattering coefficients and refractive index, were determined using an integrating sphere set-up at the Institute for Laser Technologies in Medicine and Metrology, University of Ulm.^37^^,^^38^ However, the optical properties of the four intended skin-mimicking materials could not be directly measured with the setup, as the associated absorption coefficients would exceed the measurable optical thickness over the investigated wavelength range (400 to 1000 nm). Therefore, material-mimicking-reduced pigmentation concentrations (0.0%, 0.001%, 0.003%, and 0.009% w/v) were fabricated to ensure that the sample thickness (5 mm) remained within the measurable range. Each sample was measured three times with changes in position and orientation to assess measurement repeatability. The measured absorption spectra from the four 5-mm samples were linearly scaled to the pigmentation concentrations in the skin-mimicking material used for the phantom, which yield a total absorption equivalent to that of human epidermis with a thickness of 250  μm. The optical absorption spectra of the four sulfate solutions were calculated from transmission measurements with a spectrophotometer (UV-2600i, Shimadzu, Kyoto, Japan).

The acoustic properties of the skin- and adipose-tissue-mimicking materials were measured using a modified insertion method.^39^ The setup consisted of an ultrasound (US) transmitter and a needle hydrophone positioned on opposite sides of a material sample. The needle hydrophone (SN3753 NH1000, Precision Acoustics, Dorchester, United Kingdom) was connected to a preamplifier and an oscilloscope to record signals. The US transmitter was driven by a pulser receiver (5077PR, Olympus Panametrics NDT, Waltham, Massachusetts, United States), which also provided a trigger signal to the oscilloscope. Two unfocused single-element US transducers (Olympus NDT, Waltham, Massachusetts, United States) were used, with center frequencies of 1 and 2.25 MHz (V303-SU and V306-SU, respectively). Material samples of 30×50×30  mm were prepared from the adipose-tissue-mimicking and dark skin-mimicking materials. Transmission measurements were performed through both the thick and thin sides of each sample. The speed of sound was determined from the time-of-flight of the transmitted signal, and acoustic attenuation was calculated from the amplitude reduction across the sample, following the procedure described in Dantuma et al.^39^

The density of the skin- and adipose-mimicking material was determined by measuring the volume and weight of the material samples used in the acoustic measurements. The samples were scanned using X-ray computed tomography (CT) imaging (AXIOM Artis, Siemens Healthineers, Erlangen, Germany), from which the volume was determined. The weight was determined using an analytical balance (Santorius M-power, Goettingen, Germany). The acoustic impedance was thereafter calculated from the measured density and speed of sound.

### 2.5 Phantom Fabrication

**Fig. 2:**
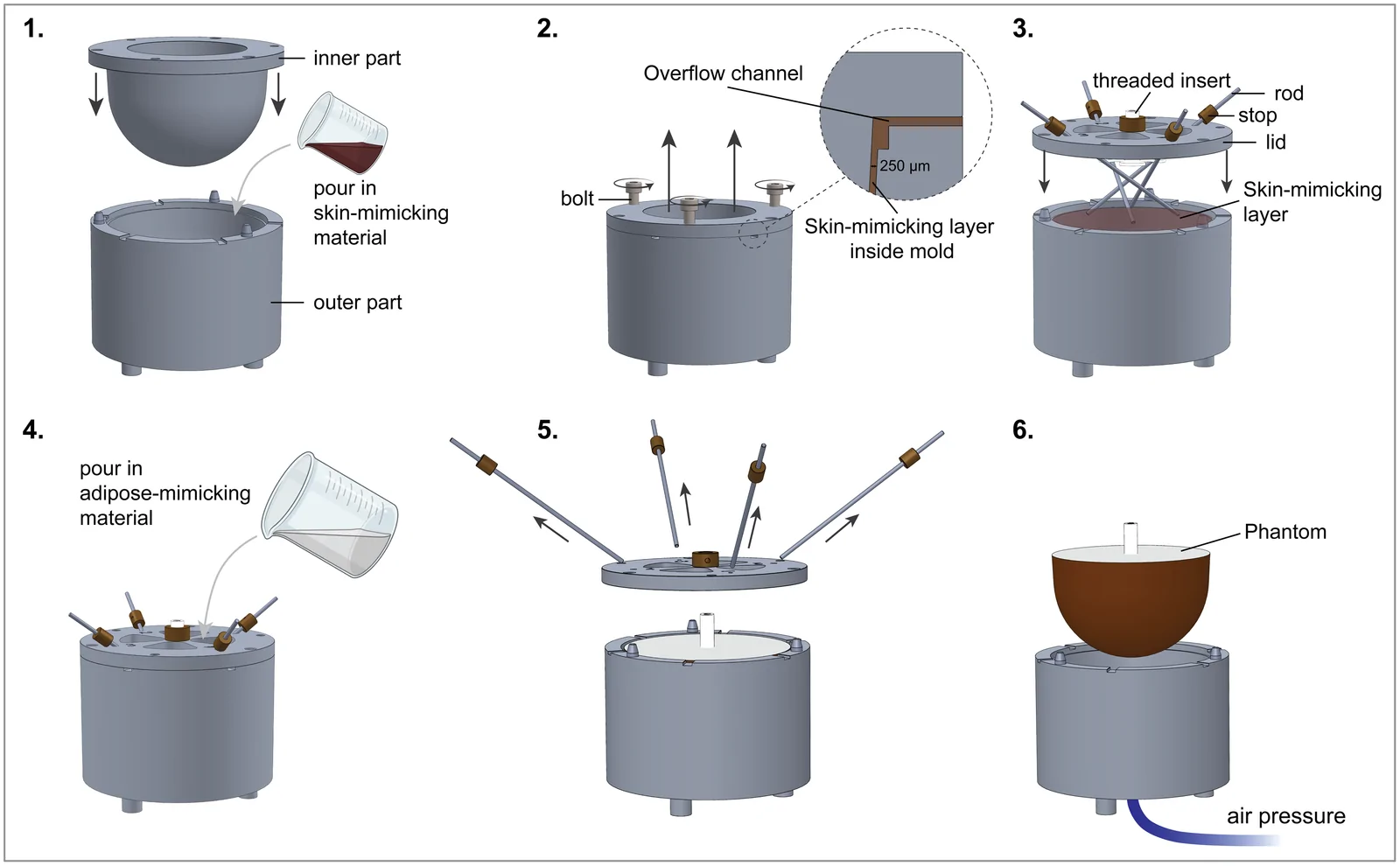
Stepwise molding procedure of the phantom. The aluminum mold consists of an inner and outer part with a 250-μm clearance defining the layer thickness. Steps include (1) casting of the skin-mimicking material, (2) cooling of the skin layer and opening the mold using bolts, (3) assembly of the lid with positioning rods and threaded insert, (4) casting of the adipose-tissue-mimicking material, (5) removal of rods and lid, and (6) demolding of the phantom.

Figure 2 illustrates the stepwise process of the phantom fabrication. CiO and NGW have melting points of 150 and 100°C, respectively, with the lower melting point of NGW ensuring compatibility with the skin-mimicking material in the two-step casting process. The phantom mold was fabricated from aluminum to withstand these high temperatures during fabrication. Computer numerical control (CNC) machining was used to achieve the fine tolerances required for the design. The mold consists of an outer and an inner part, designed with a 250-μm clearance to define the skin-mimicking layer thickness. Step 1: The outer part was coated with a silicon spray and the inner part with a thin layer of casting rubber (Shore 25, Resion, the Netherlands) to facilitate phantom removal. The mold was preheated to 150°C in an oven, and 15 mL of skin-mimicking material, at the same temperature, was poured into the outer part. The inner part was then placed on the dowel pins in the outer part to maintain position. As the mold was closed, excess material was slowly expelled through the overflow channels. Once fully closed, the mold was cooled at room temperature. Step 2: The inner part was lifted evenly using three screws, leaving a thin skin-mimicking layer in the outer part. Step 3: The lid, containing four holes for the rods and one for the 3D-printed threaded insert, was positioned on the dowel pins of the outer part. Four 3-mm-diameter metal rods and a threaded insert were placed in the lid. All components contain stops for consistent positioning. Step 4: NGW at 120°C was poured into the outer part and allowed to cool to room temperature. Step 5: All rods and the lid were removed. Step 6: Pressurized air was applied via a tube connection at the bottom of the outer part to release the phantom. The four cylindrical channels were filled with sulfate solutions with increasing optical absorption coefficient at 720 nm in clockwise order.

### 2.6 Phantom Validation

The interface between the skin- and adipose-tissue-mimicking materials was visually inspected for all phantoms. In addition, the separability of the two layers was evaluated by mechanically peeling the skin-mimicking layer to evaluate its adhesion to the adipose-tissue-mimicking material.

As the molds were fabricated using high-precision CNC machining with a dimensional tolerance of less than 2  μm, the fabrication procedure is highly repeatable and expected to yield a consistent skin layer thickness across all phantoms. To verify the dimensional accuracy of the skin layers, the non-pigmented phantom was selected as a representative sample for thickness measurements. The thickness of the skin-mimicking layer was measured using a Fourier-domain optical coherence tomography (OCT) system (Ganymede I, M00290244, Thorlabs, Newton, New Jersey, United States), equipped with a GigE camera and operating at a center wavelength of 930 nm with a bandwidth of 100 nm at 3 dB. The OCT images were acquired over a 41  mm×28  mm field of view, with lateral and axial resolutions of 4.0 and 2.7  μm, respectively. For the measurements, OCT was performed over the intact layer at three representative locations to be able to calculate the uniformity (U) of the layer, which can be calculated as follows:

$$U=1-(d_{\max}-d_{\min})/(2*\mu_{d}))*100\%,$$ (4)

where d is the layer thickness, and $\mu_{d}$ is the mean thickness. The measured optical thickness through the layer was corrected for the refractive index of the layer material determined during the material characterization (Sec. 2.4). A section of the skin-mimicking layer was peeled away, and the height of the exposed region was measured in air to verify the assumption on the refractive index of the layer material of the measurements.

To assess the structural integrity of the phantom and channels, CT imaging (AXIOM Artis, Siemens Healthineers, Erlangen, Germany) was performed to evaluate the internal structure of the phantom. The scan was made with an isotropic resolution of 0.6  mm/voxel, with the phantom fully contained within the field of view. The phantom channels were filled with air to achieve high contrast with the adipose-tissue-mimicking material.

### 2.7 Phantom Imaging

**Fig. 3:**
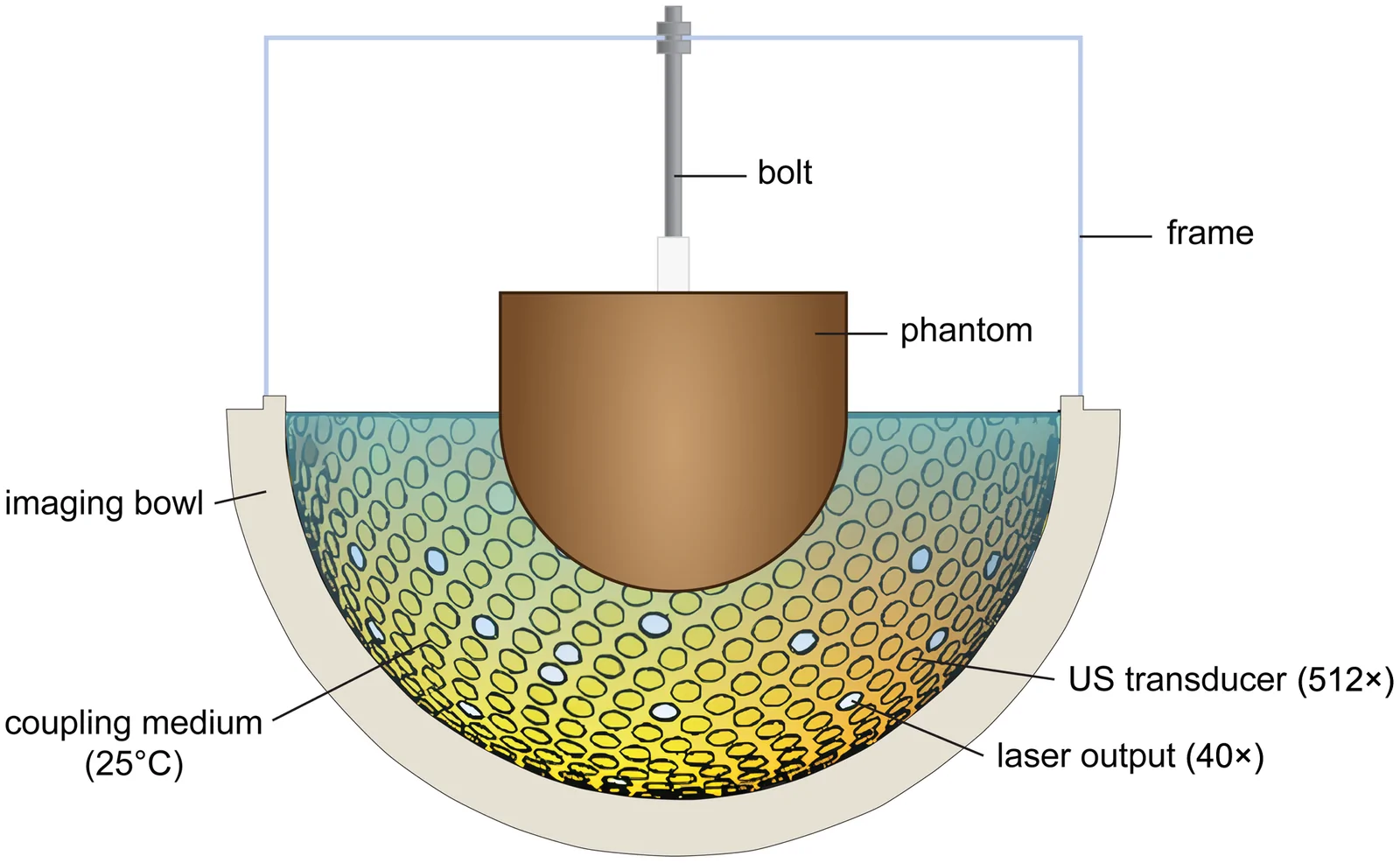
Schematic of the imaging setup using the PAM3 system. The hemispherical part of the phantom is positioned in the center of the imaging bowl. The phantom is held in place by a frame connected to its 3D-printed treaded insert.

Figure 3 shows a schematic overview of the imaging setup using the PAM3 system. The phantom was positioned in the center of the imaging bowl. A frame bolted to the phantom’s threaded insert kept the phantom in place. Water was used as a coupling medium between the phantom and the imaging bowl. Two scanning protocols were performed: (i) a full rotational scan using 10 wavelengths (680, 720, 755, 797, 833, 870, 920, 970, 1010, and 1060 nm), generating both photoacoustic and ultrasound tomography datasets, and (ii) a static scan across the full wavelength range with a 5-nm step size generating only photoacoustic data.

PA images of the full rotational scan were reconstructed using an iterative model-based reconstruction method, which uses a two-speed-of-sound model, accounting for the phantom and the surrounding water, in its wave propagation simulation.^40^ The 3D speed-of-sound maps were reconstructed from the ultrasound tomography data using a three-dimensional full-waveform inversion algorithm.^41^

### 2.8 Phantom Analysis

The signal amplitude of the skin-mimicking layer was extracted from the time-domain signals of the static scans to assess the influence of skin pigmentation, mimicked with nigrosine, on the photoacoustic intensity for different wavelengths.

The signal intensities of the channels filled with sulfate solution were extracted from the reconstructed images of the rotational scan from all phantoms, and the signal-to-background ratio (SBR) was calculated as a function of depth to evaluate the influence of skin pigmentation on imaging depth. To achieve this, the channels were first segmented using a threshold-based approach applied to the speed-of-sound maps. The segmentation masks were transferred to the photoacoustic images, and the channel location was refined by combining the local PA intensity with a 3-mm geometric constraint corresponding to the known channel diameter. The refinement step was required because the spatial resolution of the speed-of-sound image (1 mm) was lower than that of the photoacoustic image (500  μm). All processing steps were implemented in MATLAB 2025a (MathWorks, Natick, Massachusetts, United States).

The phantom surface was segmented directly from the photoacoustic images, and the minimum voxel-to-surface distance was calculated, generating a depth map in which each voxel was assigned its shortest distance to the phantom surface. A sagittal cross-section of the depth map is shown in Fig. S1 in the Supplementary Material. The phantom volume was divided into 3-mm depth intervals. For each interval, the mean photoacoustic intensity within each channel was calculated using only voxels exceeding the −3-dB threshold relative to the maximum intensity inside the channel at that depth to exclude noise.

The background intensity for each interval was calculated from the voxels outside the channel mask. A 3-mm exclusion margin was applied around each channel to avoid contributions to the background intensity from channel-induced artifacts. The SBR for each depth interval was calculated as

$$\mathrm{SBR}=10{\;\log}_{10}(\frac{S}{B}),$$ (5)

where S is the mean channel intensity above the −3-dB threshold per depth interval, and B is the corresponding mean background intensity.

## 3 Results

### 3.1 Tissue-Mimicking Material Characterization

**Fig. 4:**
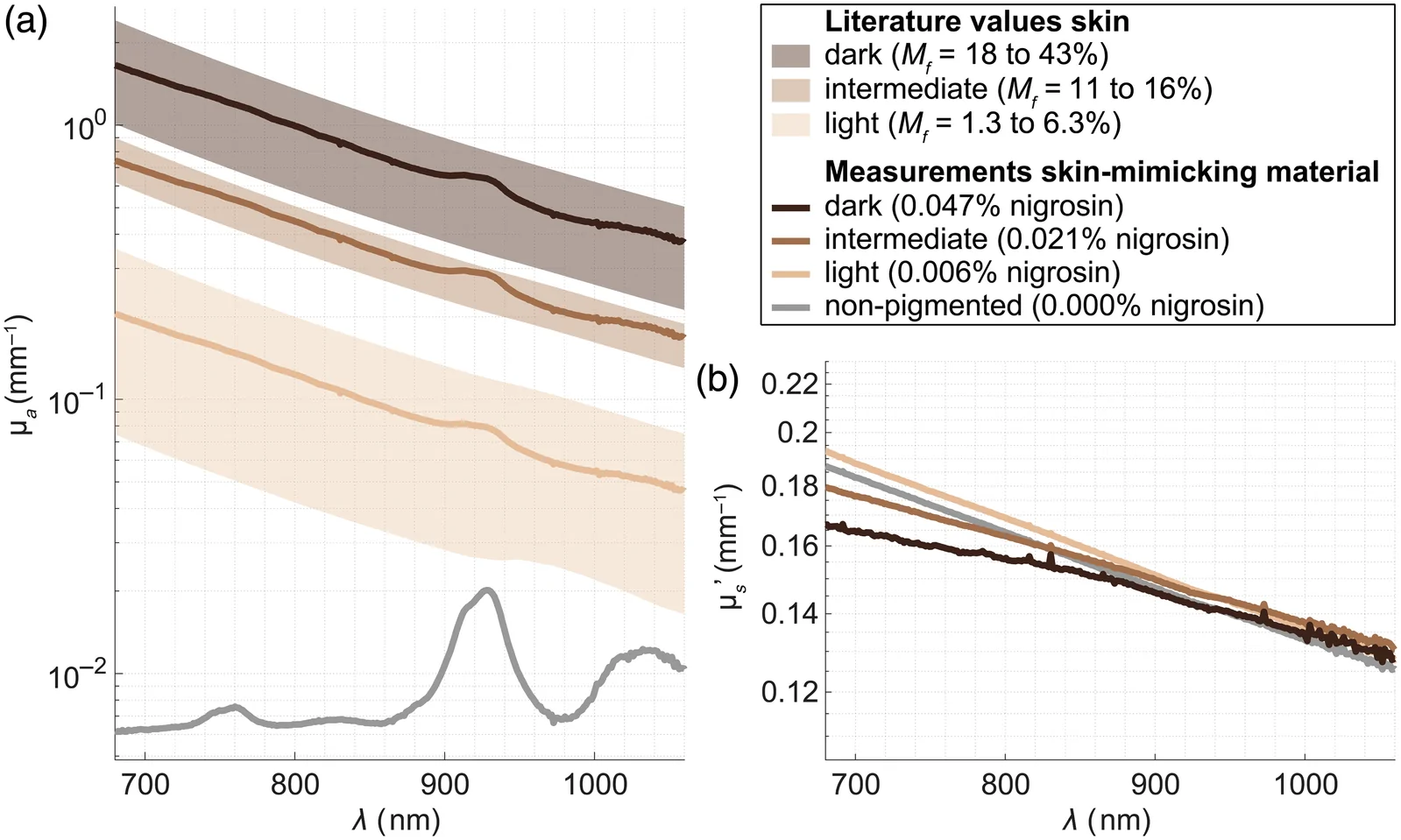
(a) Absorption ($\mu_{a}$) and (b) reduced scattering coefficient ($\mu_{s}^{\prime }$) of the skin-mimicking material in the phantom (copolymer-in-oil with nigrosine) for four skin pigmentation levels: non-pigmented, light, intermediate, and dark skin. The absorption spectra are linearly scaled from the measured spectra of the samples with reduced nigrosine concentrations. The literature-derived absorption spectra [based on Eqs. (2) and (3)] from the epidermal layer with the relevant Mf value ranges were shown for comparison. To match the total optical absorption between experimental and literature values, the absorption coefficients of the literature values were scaled to the thicker skin-mimicking layer.^25^

Figure 4 shows the optical properties of the skin-mimicking materials used for the four skin-mimicking phantoms. These spectra were corrected to account for the reduction in the nigrosine concentration in the measured samples. The absorption spectra are in good agreement with the literature-derived spectra from the epidermis across the full wavelength range of 400 to 1000 nm, when scaled to the same thickness. The reduced scattering coefficients remained nearly constant across all pigmentation levels, indicating that scattering was unaffected by changes in nigrosine concentration. Repeated measurements showed minimal variability in the optical properties of the skin-mimicking materials across all samples.

**Fig. 5:**
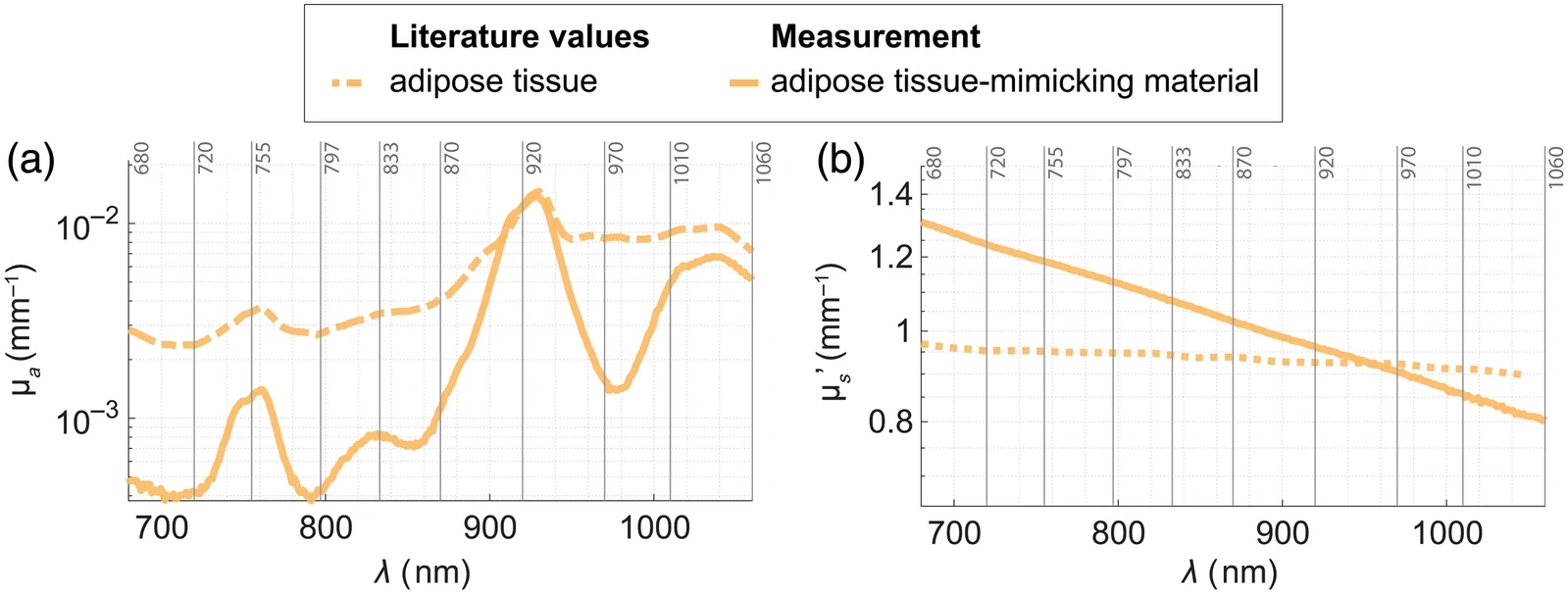
(a) Absorption ($\mu_{a}$) and (b) reduced scattering coefficient ($\mu_{s}^{\prime }$) of the adipose-mimicking material (native gel wax with additives) compared with the literature values of adipose breast tissue.^21^ The vertical solid lines indicate the wavelengths used during imaging.

Figure 5 shows the optical properties of the adipose tissue-mimicking material compared with the literature values of adipose breast tissue.^21^ In the measured spectra, two peaks are observed at 755 and 920 nm, which can also be seen in the spectrum of adipose breast tissue. The peak at 755 nm is slightly lower than the literature value, whereas the peak at 920 nm closely matches that of adipose tissue. The measured reduced scattering spectrum decreases from 1.3 to $0.8\;{\mathrm{mm}}^{-1}$ across the wavelength range of 400 to 1000 nm, which is close to the desired constant value of $1\;{\mathrm{mm}}^{-1}$ for adipose tissue.

**Fig. 6:**
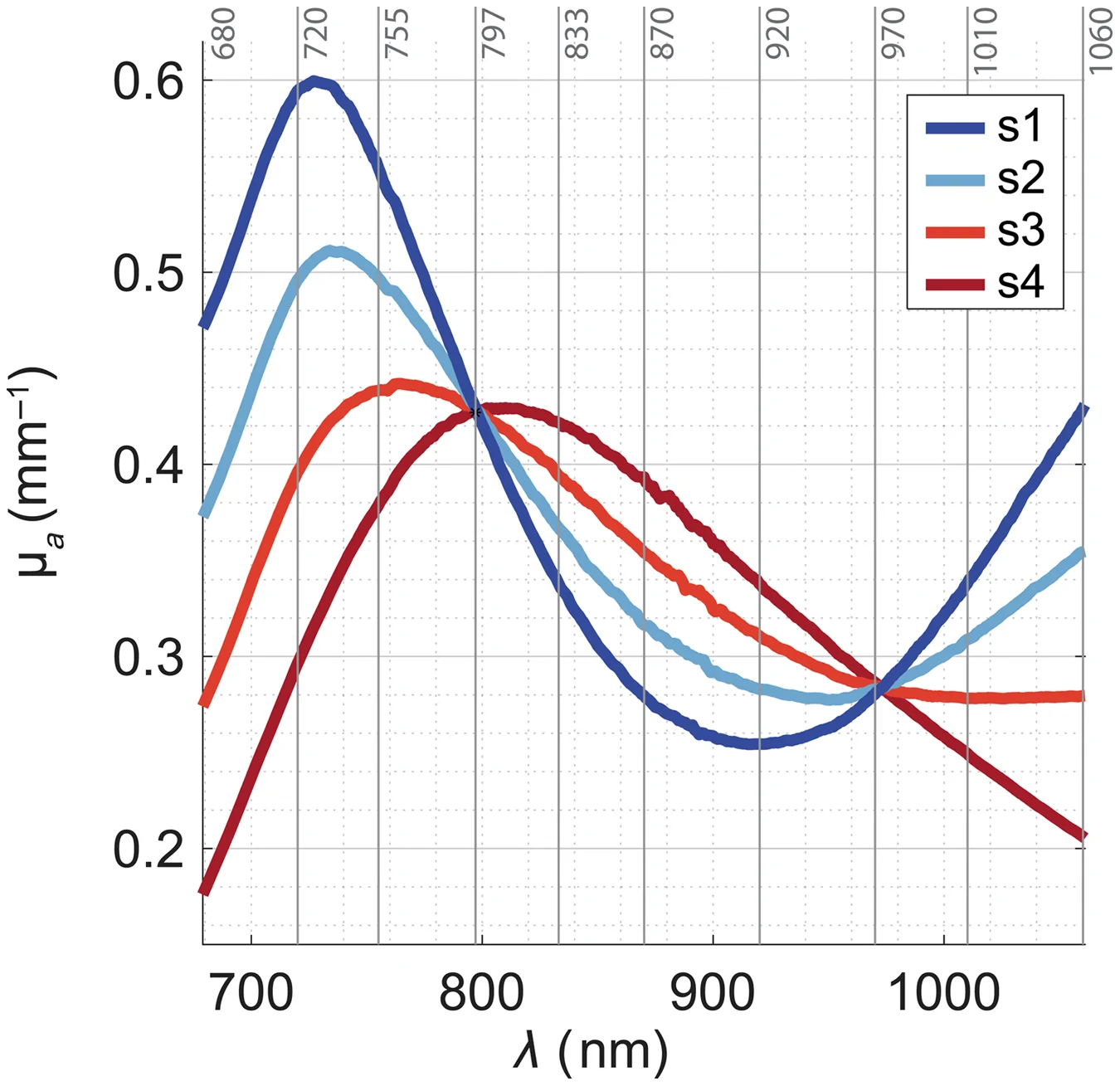
Absorption coefficient ($\mu_{a}$) of the four sulfate solutions (s1 to s4). The vertical solid lines indicate the wavelengths used during imaging.

Figure 6 shows the absorption spectra of the four sulfate solutions. As the copper sulfate concentration decreases, the peak at 730 nm increases, whereas the minimum at 920 nm decreases, consistent with the trend reported by Fonseca et al.^33^ As per design, an isosbestic point is observed at 797 nm, with an absorption coefficient of $0.427\;{\mathrm{mm}}^{-1}$, matching the absorption coefficient of the blood ($0.43\;{\mathrm{mm}}^{-1}$) at a hemoglobin concentration of 150  g/L.^36^

The measured acoustic properties are summarized in Table 1. The speed of sound in the skin-mimicking material was 1477  m/s, which is lower than that of the epidermis, but well within the range of soft tissue (1450 to 1575  m/s). The acoustic attenuation was 0.499  dB/cm at 1 MHz, which is well within the range of the epidermis. The speed of sound (1441  m/s) and acoustic attenuation at 1 MHz (0.585  dB/cm) of the adipose tissue-mimicking material are in good agreement with literature values of adipose tissue. The calculated acoustic impedance of both skin- and adipose tissue-mimicking is 1.23 MRayl, resulting in no acoustic reflection between both materials.

**Table 1:** The measured speed of sound ($c_{s}$), frequency-dependent acoustic attenuation ($a_{s}$), density (ρ), and acoustic impedance (Z) of the skin- and adipose-tissue-mimicking materials together with literature values. The literature values show the range from the different studies.

	$c_{s}\mathrm{(m/s)}$	$\alpha_{s}\mathrm{(dB/cm)}$	$f{\left(\mathrm{MHz}\right)}^{n}$	${\rho (\mathrm{kg/m}}^{3})$	Z (MRayl)	References
Epidermis	1645 ± 42	0.44 ± 0.26*f*	1.55 ± 0.12	1150	1.53 to 1.68	31 and 42
Skin-mimicking material	1477 ± 1	0.499*f*	1.807	835	1.23	
Adipose tissue	1455 ± 25	0.158 to 2.0*f*	1.7 to 2.0	937	1.33 to 1.39	32, 43, and 44
Adipose-mimicking material	1441	0.585*f*	1.582	855	1.23	

### 3.2 Phantom Validation

**Fig. 7:**
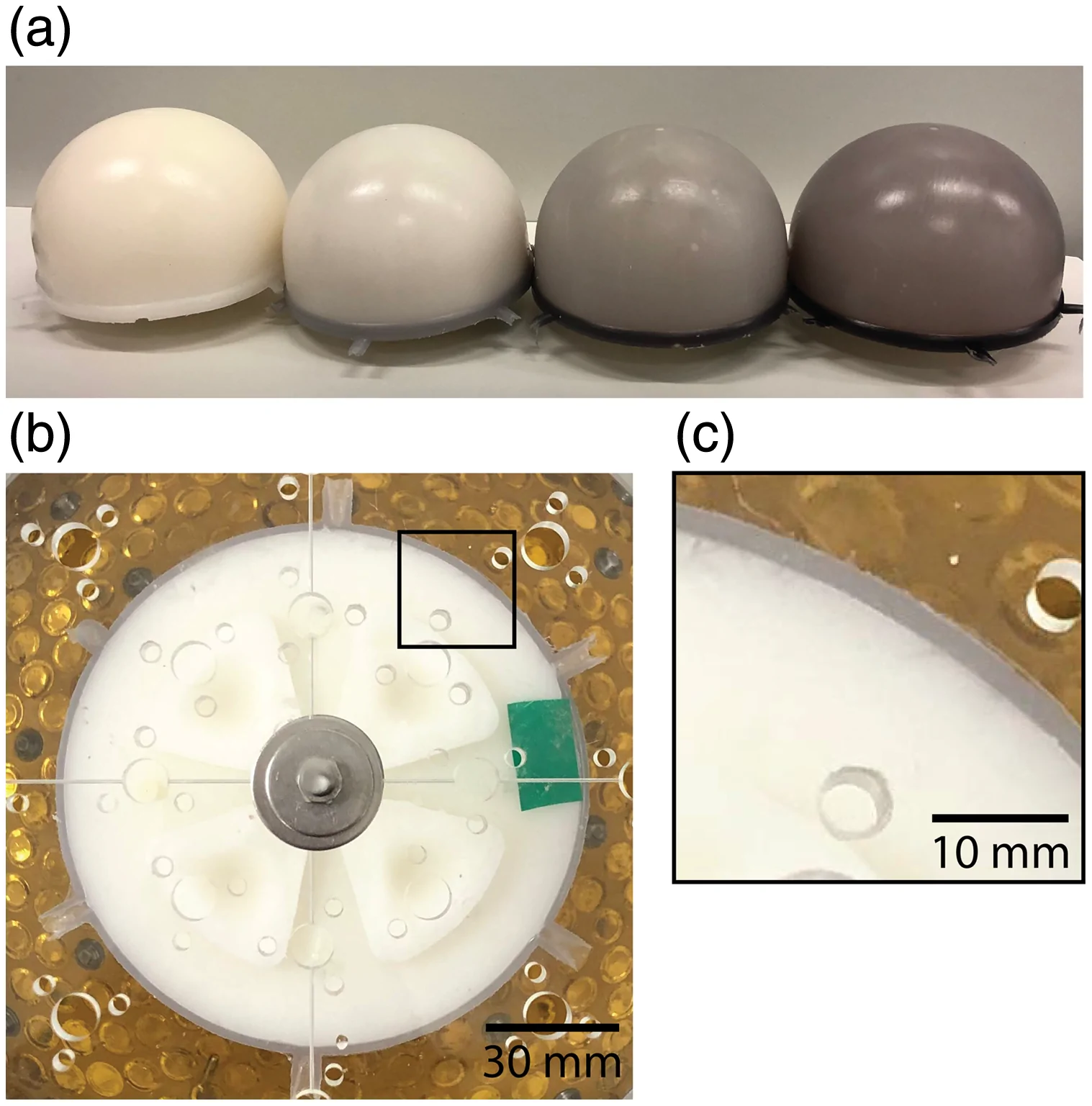
Photograph of the skin color phantoms. (a) The four phantoms are arranged from left to right: non-pigmented, light, intermediate, and dark skin. (b) Top view of the light skin phantom positioned in the PAM3 system. (c) Zoom in on the skin layer of the phantom. Gray streaks at the surface are surface artifacts (burrs) and roughness caused by the removal of the mold and do not indicate diffusion of nigrosine into the bulk phantom.

Figure 7 shows a photograph of the four fabricated phantoms, arranged from left to right in order of increasing skin-mimicking pigmentation. The pigmentation levels correspond to nigrosine concentrations of 0%, 0.006%, 0.021%, and 0.047% w/v for non-pigmented, light, intermediate, and dark skin, respectively. In Fig. 7(b), a top-view photograph of the light-skin phantom in the PAM3 system is shown. The boundary between the skin-mimicking and the underlying adipose-mimicking layers is clearly visible, confirming that the layered structure remains intact after fabrication, and the nigrosine does not diffuse into the adipose-tissue-mimicking material. The non-pigmented phantom was subsequently sectioned, and the skin layer could be cleanly peeled off from the adipose layer, further verifying the distinct layer interfaces.

**Fig. 8:**
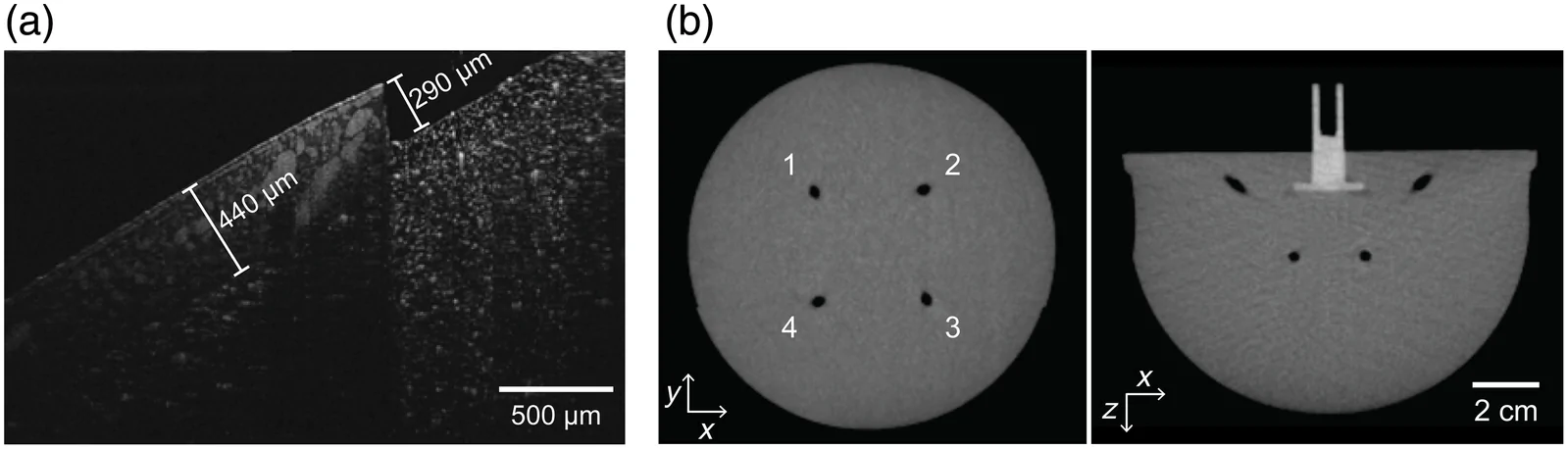
OCT and CT image of the non-pigmented phantom. (a) OCT image of the skin-mimicking layer, including an optical thickness of 440  μm and an air measurement of a stripped section of 290  μm. (b) Axial and coronal CT image of the phantom, including four channels of 3 mm and a threaded insert.

Figure 8(a) shows an OCT cross-section of the skin-mimicking layer of the non-pigmented phantom. The optical layer thickness measured by OCT was 440±20  μm. After applying the refractive index correction of the material (n=1.47), the physical layer thickness was calculated to be 299  μm. For verification, a section of the layer was peeled off and measured in air, yielding a thickness of 290  μm. This measurement is in close agreement with the OCT-derived thickness through the layer (3% difference). The measured thickness through the layer approaches the 250-μm layer thickness defined by the mold (16% difference) and shows uniformity of 95% over the layer.

Figure 8(b) shows the CT images of the non-pigmented phantom in the axial and coronal planes. No contrast is observed between the skin-mimicking and adipose-tissue-mimicking material, indicating a good adhesion between the two layers. In addition, no air inclusions were detected either at the interface between the two materials or within the bulk adipose material. The four cylindrical channels are clearly visible, intact, and match the intended diameter of 3 mm.

### 3.3 Phantom Analysis

#### 3.3.1 Skin Signal Intensity

**Fig. 9:**
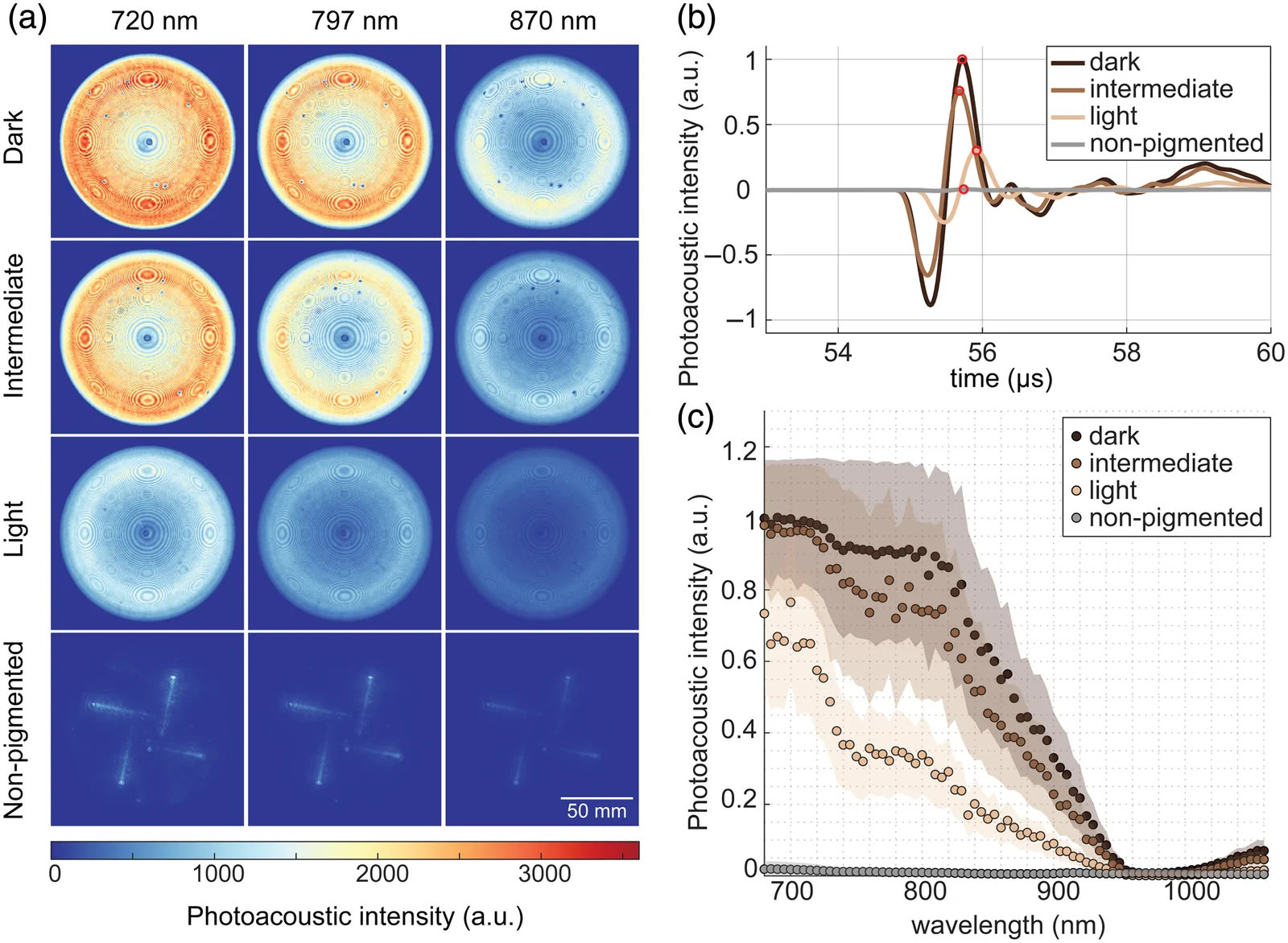
Photoacoustic signals from the skin-mimicking layer. (a) MIPs of the reconstructed photoacoustic images of the four phantoms in coronal view at three wavelengths: 720, 797, and 870 nm. (b) Time-domain signals measured by the bottom transducer at 830 nm for all phantoms. Red markers indicate the maximum peak of each time signal. Normalized photoacoustic signal intensity of the skin-mimicking layer across the full wavelength range (680 to 1060 nm). (c) Normalized photoacoustic signal intensity of the skin-mimicking layer across the full wavelength range (680 to 1060 nm).

Figure 9(a) presents the maximum intensity projection (MIP) of the reconstructed photoacoustic images of the four phantoms in coronal view for three wavelengths: 720, 797, and 870 nm. A strong dependence of the skin signal intensity on the concentration of nigrosine in the skin-mimicking layer is observed. The dark-skin phantom consistently exhibits the highest skin signal at all wavelengths, followed by the intermediate- and light-skin phantoms. The non-pigmented phantom showed negligible signal intensities from the skin-mimicking layer. As a result, the channels filled with sulfate solution are clearly visible in the MIP of the non-pigmented phantom, whereas in the other phantoms, they are obscured by the strong skin signal.

Figure 9(b) shows the photoacoustic time signals detected by the bottom transducer at 830 nm for all phantoms. The peak amplitudes of these signals were extracted to quantify the photoacoustic intensity of the skin layer. Figure 9(c) presents the normalized peak intensities of the skin-mimicking layer across the full wavelength range (680 to 1060 nm) obtained from the static scans. The values decrease monotonically with increasing wavelengths up to 920 nm, beyond which the optical absorption of water increases. A plateau is observed between 750 and 790 nm, consistent with previously reported *in vivo* measurements.^18^ In the dark- and intermediate-skin phantoms, the signals were clipped for the majority of the transducers in the 680 to 710 nm range due to the high pressure generated by the skin layer, resulting in increased standard deviation. Acquisition settings were optimized to preserve sensitivity to weaker signals at depth. As a result, clipping affects only superficial signals, and signals from deeper-laying structures remain within the measurable range and are used for analysis.

#### 3.3.2 Imaging Depth

**Fig. 10:**
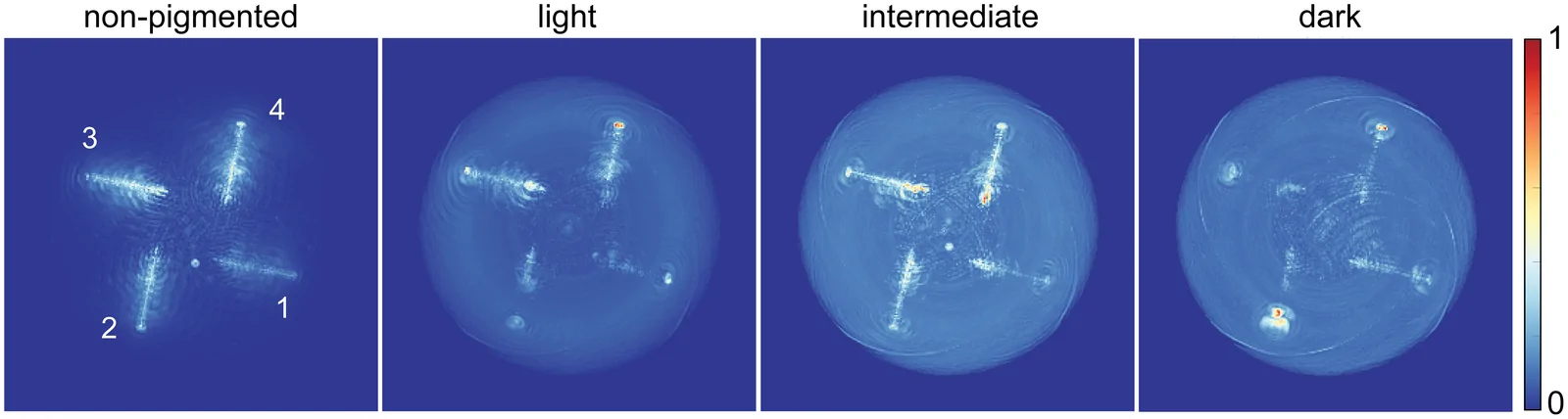
Depth-weighted MIPs of the reconstructed photoacoustic images of the four phantoms in coronal view at 720 nm. Channels 1 to 4 contain sulfate solution s1 to s4.

Figure 10 shows the depth-weighted MIP of the photoacoustic reconstructions for all four phantoms at 720 nm. The channels filled with sulfate solution are visible in all images; however, their visibility decreases with increasing pigmentation. Interestingly, a slightly higher channel signal is observed in the intermediate skin phantom compared with the light phantom. The background signal increases from the non-pigmented to the dark phantom, due to skin-layer photoacoustic signals reflecting at the phantom-water interface, appearing as diffuse background or curved artifacts within the phantom. Reflection artifacts from the channels are also observed, and additional artifacts might have arisen from the reconstruction algorithm. Close-up views of the artifacts are shown in Fig. S2 in the Supplementary Material.

The SBR was calculated from the reconstructed image for the channel filled with the solution corresponding to the highest absorption coefficient (s1) at 720 nm, among the four solutions. The depth-dependent channel signal intensity is highest in the non-pigmented phantom, followed by the intermediate, light, and dark phantoms (Fig. S3 in the Supplementary Material). In the non-pigmented phantom, the background signal increases gradually with depth, whereas in the light, intermediate, and dark phantoms, it fluctuates. These fluctuations spatially correspond to the position of the reflection artifacts caused by the skin-mimicking layer. Absolute signal amplitudes are influenced by variations in laser pulse energy. To account for this, the data were normalized, and results are reported in terms of relative metrics such as SBR, which are insensitive to global scaling of excitation energy. This ensures that the observed trends reflect differences in optical absorption rather than variations in laser output.

**Fig. 11:**
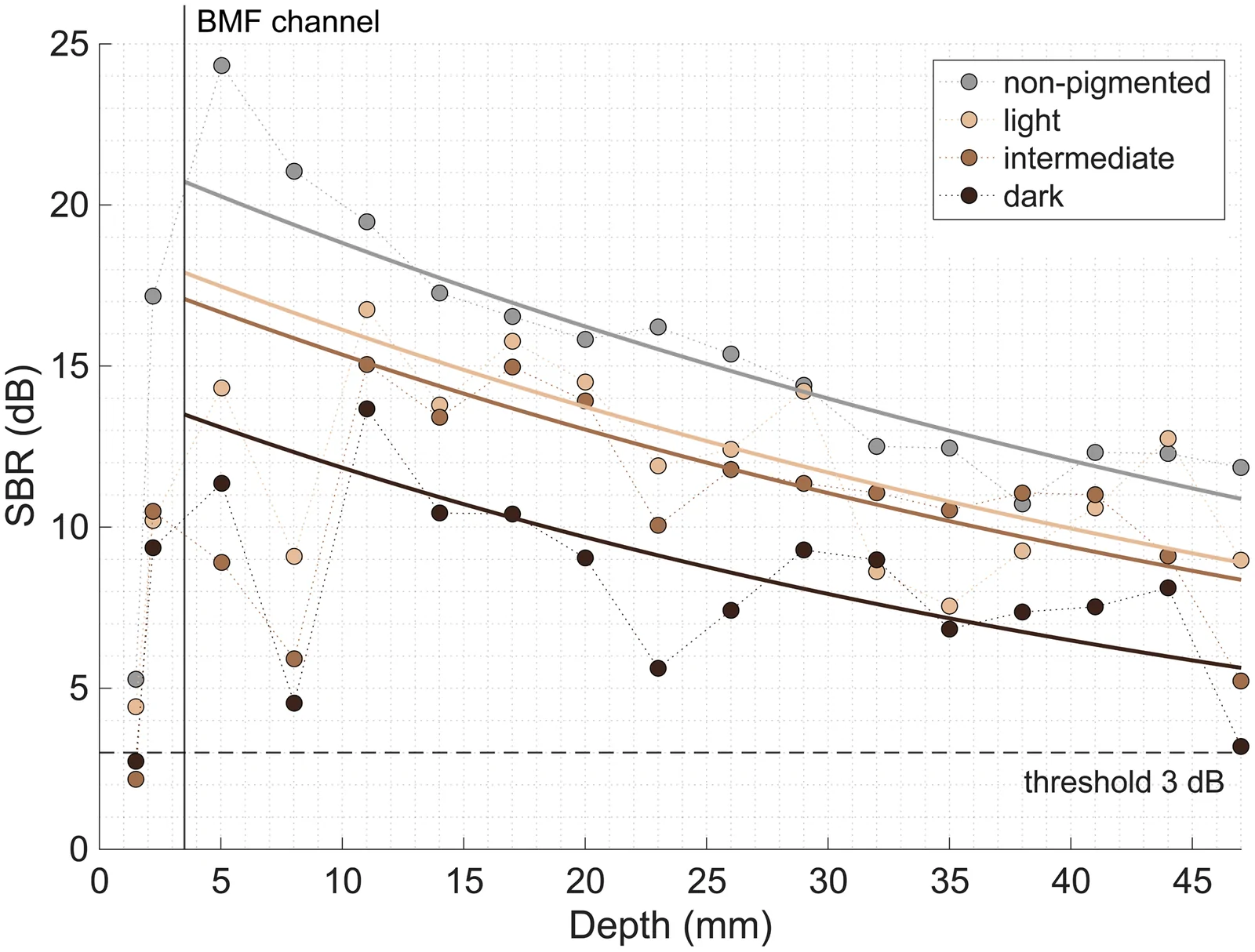
SBR as a function of depth for channel filled with s1 at 720 nm for all four phantoms. Dots represent the measured values, and lines show the fitted exponential curves of the channels. The dashed line is provided as guide to the eye. The channel starts from 3.5 mm below the phantom surface, as indicated by the vertical black line.

The resulting SBR as a function of depth is shown in Fig. 11. The channel starts from 3.5 mm below the surface. In all phantoms, the SBR decreases with depth and is consistently reduced as skin-mimicking pigmentation increases. In the non-pigmented phantom, the SBR exhibits an exponential decay with increasing depth. In contrast, the light, intermediate, and dark phantoms display multiple local minima rather than a monotonic decrease. These dips coincide with fluctuations in background signal intensity (Fig. S3 in the Supplementary Material), and this appears to result from the reflection-related artifacts discussed earlier, which locally reduce the SBR.

#### 3.3.3 Spectral behavior

**Fig. 12:**
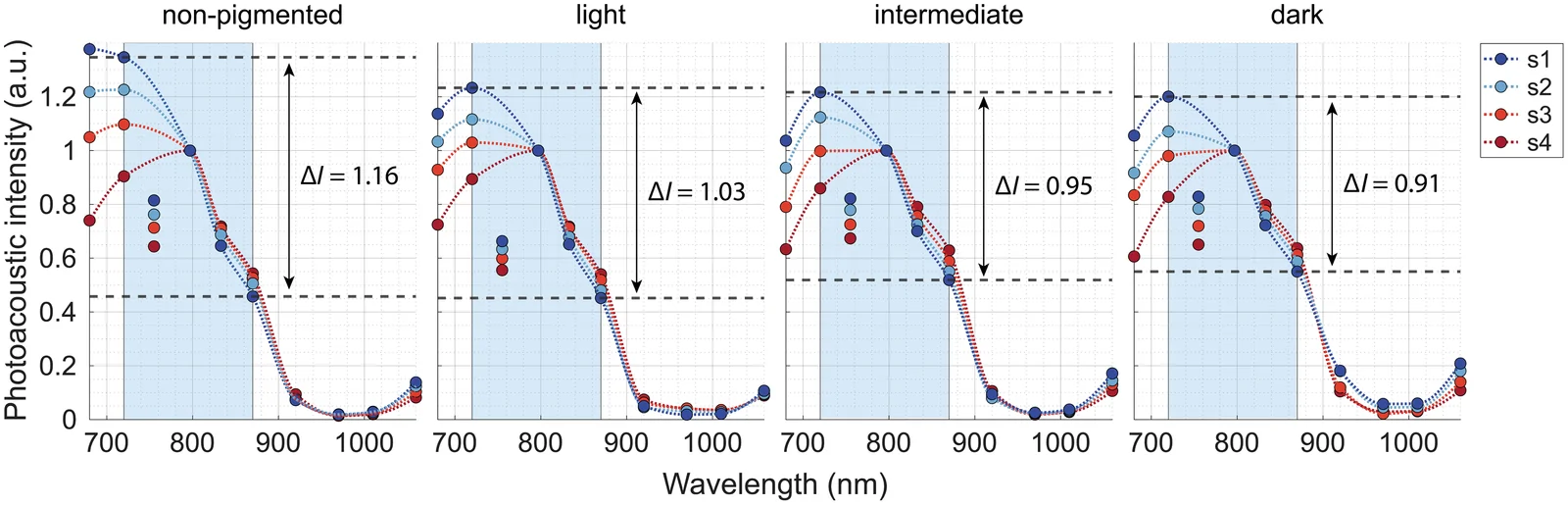
Mean photoacoustic intensity of all four channels (s1 to s4) as a function of wavelength for the 25- to 35-mm depth interval. Signals are normalized to the isosbestic point at 797 nm, corrected for the laser power, and no fluence correction is applied. The region of interest (720 to 870 nm) is shown by the blue-shaded area. ΔI shows the difference between the minimum and maximum intensity in the region of interest.

Figure 12 shows the mean photoacoustic intensity from the four channels as a function of wavelength for the 25 to 35 mm depth interval, which exhibits minimal interference from reflection artifacts. The signals were corrected for laser output power and normalized to the isosbestic point at 797 nm. No fluence correction was applied; therefore, no direct comparison can be made with the characterized spectra of the sulfate solutions (Fig. 6). All spectra show a significant decrease in intensity beyond 870 nm, corresponding to the increasing absorption of water in the imaging bowl. A local minimum is observed at 980 nm, corresponding to a strong water absorption band that reduces the fluence available for photoacoustic signals at this wavelength.

Wavelengths of interest are highlighted in Fig. 12 by a blue shaded area (720 to 870 nm). This wavelength range exhibits the most significant spectral spread relative to the isosbestic point. Moreover, it corresponds to the region that provides the strongest chromophore-dependent contrast. The spectral behavior in this region is consistent with the independently characterized absorption spectra of the solutions, particularly in the reversal of the relative intensity order after the isosbestic point. The observed deviations at 755 nm cannot be attributed to the absorption spectra of the solutions. A similar irregularity was observed in the intensity spectrum of the skin-mimicking layer [Fig. 9(b)]. Although water shows a weak absorption around 760 nm, this is not likely to account for the substantial deviation. Therefore, the 755-nm values were excluded from interpretation.

Across phantoms, the intensity difference in the wavelengths of interest (720 to 870 nm) decreases with increasing skin-mimicking pigmentation. For the channel filled with s1, the intensity difference ($\Delta I=\max(I_{720})-\min(I_{870})$) is 1.16, 1.03, 0.95, and 0.91 for the non-pigmented, light, intermediate, and dark phantom, respectively. The trend is consistent with increased absorption of the skin-mimicking layer at shorter wavelengths in more pigmented phantoms, reducing the local fluence and disproportionately attenuating the signal intensity from the channels at the wavelengths of interest.

## 4 Discussion

We have developed breast phantoms with a skin-mimicking layer to evaluate the effect of skin pigmentation on PA tomography imaging. Four phantoms with identical geometry and material composition were fabricated, differing only in pigmentation level of the skin-mimicking layer, ranging from non-pigmented to light, intermediate, and dark skin tones. By imaging the phantoms using the PAM3 system, we were able to identify and quantify the impact of skin pigmentation on the PA imaging performance.

The phantom materials demonstrated the required optical and acoustic properties for the intended phantom design, consistent with values reported in the literature. In particular, the optical absorption of the skin-mimicking layer closely aligns with epidermal reference data. A key factor appears to be the dissolution of nigrosine in ethanol prior to incorporation into the copolymer-in-oil mixture, which produces absorption spectra that closely resemble those of the native dye. This contrasts with the approach of Hacker et al.,^23^ who added nigrosine directly in its pure powder form to the copolymer-in-oil mixture, resulting in absorption spectra that deviated from those of the native dye. Although the shelf life of the assembled phantoms was not explicitly evaluated in this study, all materials have been reported to exhibit long-term stability, suggesting that fabricated phantoms are suitable for extended use.^23^^,^^29^

Phantom fabrication was shown to be consistent and met the design specifications. Visual inspection revealed a clear boundary between the skin- and adipose-tissue-mimicking materials, and the ability to peel off the skin-mimicking layer indicates that no unintended material merging occurred. OCT imaging in the non-pigmented phantom showed that the skin-mimicking layer was 290  μm thick (16% thicker than the targeted 250  μm) and demonstrated that the fabrication procedure reliably produced a thin, uniform layer. This measurement was considered representative for the other cases, as OCT imaging could not be performed through the pigmented layers due to their opacity. Furthermore, no variability during fabrication was observed: in all phantoms, the mold consistently closed completely, excess material was expelled through the overflow channels, and the cavity for the skin-mimicking layer was fully filled. Therefore, minimal variability among the layers is assumed. Although the layer is slightly thicker than intended, it is still below the resolution of the PAM3 system (400  μm) and, therefore, satisfactory for the purpose of the study. In addition, in this study, we were interested in relative changes in PA intensity values rather than absolute values; therefore, uniformity of this skin layer (variation within 4.5%) is more critical than layer thickness. CT imaging showed that the four channels in the phantom were clearly visible, intact, and free of air bubbles. No air inclusions were observed elsewhere in the phantom either.

Our 3D phantom enables simultaneous visualization of multiple sulfate solutions with depth-resolved information, allowing several chromophore concentrations to be assessed at various depths in a single measurement. In contrast, most recently developed phantoms that study the effect of skin color using 2D imaging systems contain only one or multiple channels at different depths, using a single dye or swapping dyes among channels to compare chromophore spectra, due to the limited field of view of these systems.^16^^,^^17^ By measuring both spectral and depth information in a single scan, our phantom provides directly comparable data that more closely reflect *in vivo* conditions.

The SBR of the channels filled with sulfate solution decreases with depth as skin pigmentation increases. This measured trend is consistent with observations from previous phantom and *in vivo* studies using 2D systems, where increasing skin absorption reduces fluence at depth.^7^^,^^10^^,^^17^ Although more difference between the SBR of the light and intermediate pigmentation phantoms in our study was anticipated, the reduced discrimination may be attributable to an unintended decrease in the laser output power of the PAM3 system. Because the primary focus of this study is on relative trends rather than absolute SBR values, this limitation is not expected to affect the main conclusions.

Artifacts observed in the reconstructed images were predominantly reflections from the phantom surface and were more pronounced with increasing pigmentation. This is expected, as stronger skin optical absorption produces higher surface signals, resulting in stronger reflection artifacts at the phantom-water interfaces due to the impedance mismatch (R=11%) between the phantom materials and the water in the imaging bowl. Additional reflection artifacts were observed around the channels due to similar impedance mismatches. Impedance mismatches are a known limitation of simplified phantoms, but maintaining minimal complexity was necessary to isolate the impact of skin pigmentation. Nonetheless, these artifacts do not affect our observations that increased pigmentation levels reduce imaging depth. They had an influence on the SBR values at individual depths but did not affect the overall trend. In contrast, *in vivo* breast imaging typically shows fewer reflection artifacts due to smoother acoustic transitions among soft tissues, and the acoustic impedance of the skin more closely matches that of water.^45^

Although several studies report clutter artifacts dominating at higher pigmentation levels,^7^^,^^10^ clutter artifacts were minimal in our tomographic measurements. Although pigmentation increased the amplitude of reflection-induced background signals, they did not overlay the channels and therefore did not interfere with detectability. This suggests that tomography-based acquisition and 3D reconstruction may inherently reduce skin pigmentation-induced clutter compared with 2D imaging approaches.

Spectral measurements from the four sulfate solutions showed that the relative intensity difference (ΔI) at the wavelengths of interest (720 to 870 nm) decreases with increasing pigmentation levels. This trend is consistent with the higher absorption by the skin-mimicking layer at shorter wavelengths, leading to a spectral coloring effect. The decrease in ΔI with increasing pigmentation levels showed that it becomes more difficult to discriminate among different chromophore concentrations based on their PA intensity spectra at the wavelength of interest. This trend indicates that, in this region, discrimination of chromophores, such as oxy- and deoxyhemoglobin, would become increasingly difficult as pigmentation levels increase, which would directly propagate into errors in the estimation of chromophore concentrations. This reduced spectral contrast will lead to higher variance of estimate, decreasing the reliability of the measurements in darker skin. In addition, if no calibration for skin pigmentation is applied, this will ultimately lead to biased estimates of physiological parameters such as oxygen saturation. Previous phantom and *in vivo* studies using 2D PA imaging systems reported pigmentation-related biases in oxygen saturation measurements.^16^^,^^17^ However, this is the first time that pigmentation-induced alterations in measured spectra have been demonstrated and directly linked to chromophore concentration bias in a PA tomography setting, which is the basis for oxygen saturation measurements.

Although the breast phantom developed in this study represents a simplified model of human breast tissue, the results demonstrate strong consistency with previously reported *in vivo* measurements obtained using 2D PA imaging systems.^7^^,^^9^^,^^10^ Several factors should be considered when interpreting the findings of this study. The epidermis-mimicking layer (290  μm) was thicker than the human epidermis (≈77  μm).^20^ Although the absorption coefficient of this layer was scaled to match the absorbance of the real epidermis, the increased thickness alters the spatial distribution of absorption. Equivalent absorbance in a thinner layer would generate higher acoustic frequencies, which are stronger attenuated and fall largely outside the detection bandwidth of the PAM3 transducers (1 MHz, 123%). As a result, the system is primarily sensitive to the integrated absorption (i.e., total absorbance), rather than its precise spatial distribution within the thin layer. In addition, the straight channels incorporated into the phantom do not fully capture the geometric complexity of real vascular networks, and the sulfate solutions do not reproduce the optical properties of human blood.^46^ Despite these limitations, the agreement among phantom measurements and previously reported *in vivo* data indicates that the dominant effect of melanin-driven effects is captured in the present studies.

This work presents an important step toward the quantitative assessment of pigmentation bias in PA tomography. The phantom, which was technically challenging to develop, is well documented, and the fabrication method provides reproducible, well-characterized phantoms with controlled, relevant features. This phantom is highly suitable for systematically studying the effects of skin pigmentation and for developing and evaluating correction strategies to mitigate skin pigmentation-related bias in quantitative chromophore concentration measurements. By incorporating controlled bulk material optical properties, this fabrication approach can be extended to develop advanced phantoms for investigating spectral coloring and quantitative imaging,^35^ which is one of the key challenges in the field.

The fabrication procedure for the phantom is adaptable and straightforward. It can therefore also be used to develop photoacoustic tomography setups with other geometries, providing flexibility across imaging systems.^47^^–^^51^ As such, this phantom fabrication approach has the potential to become widely applicable for benchmarking PA performance under controlled yet clinically relevant conditions.

## 5 Conclusion

Our study shows that PA tomography is susceptible to pigmentation-related bias: increasing skin optical absorption reduces signal-to-background and distorts spectral measurements, which can lead to systematic errors in chromophore concentration estimates. These effects were systematically investigated using breast phantoms with identical geometry but varying levels of skin-mimicking pigmentation. The phantoms incorporate a method for fabricating a thin, uniform skin-mimicking layer on a hemispherical surface, and verification measurements confirmed that the design specifications were met. The phantom materials were fully characterized and closely matched the optical and acoustic properties of skin and adipose tissue. These well-characterized and validated phantoms provide a controlled platform for studying the effects of skin pigmentation and for developing strategies to improve the reliability and inclusivity of photoacoustic tomography imaging across skin tones.

## Supplementary Material

10.1117/1.BIOS.3.2.022510.s01

## Data Availability

Open source code: github.com/riannebulthuis/SkinColorPATPhantom.
