# Supplementary material for: Impact of skin pigmentation on photoacoustic tomography: a phantom study
Source: Biophotonics Discov. 2026 Jun 3;3(2):022510. doi: 10.1117/1.BIOS.3.2.022510 (PMC13559163; doi:10.1117/1.BIOS.3.2.022510)
Supplement: Supplementary file 1 [file BIOS_003_022510_SD001.pdf]

Supplementary Material

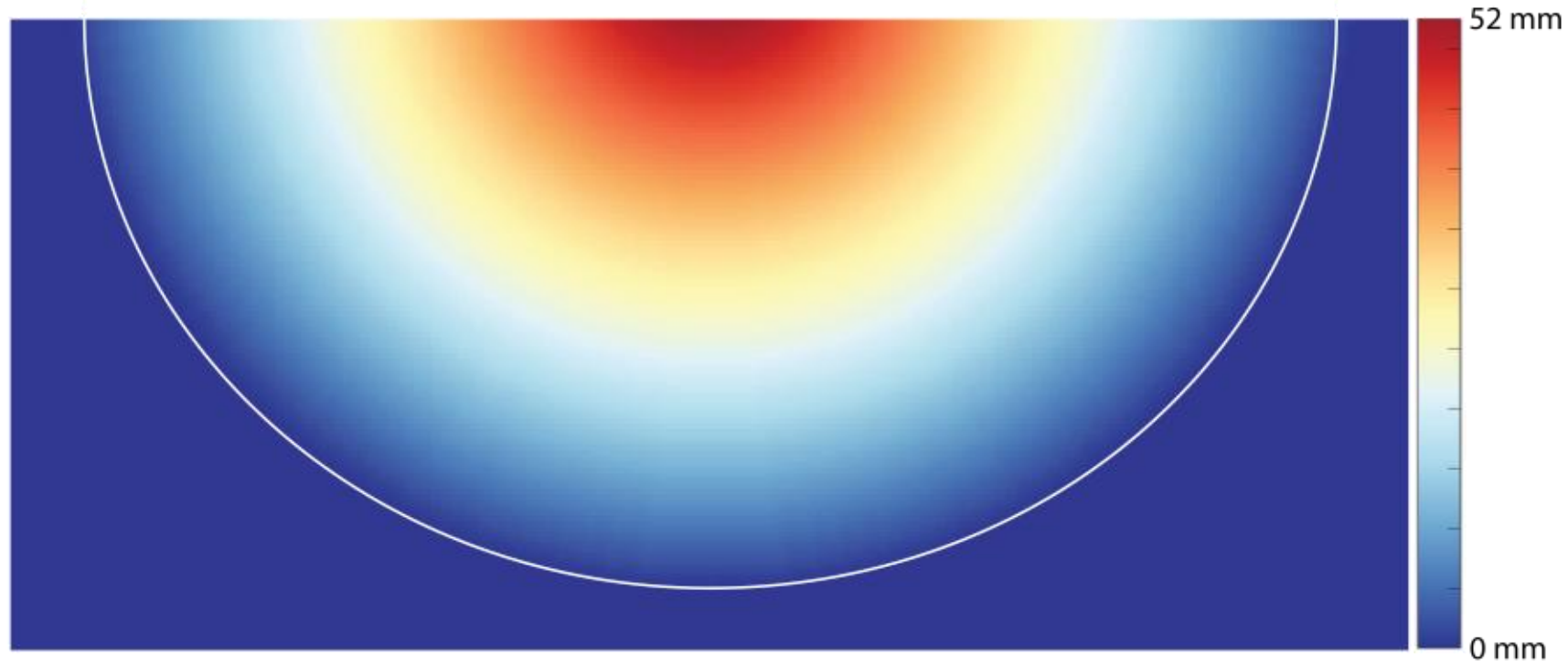

Figure S1 Sagittal cross-section of the depth map.

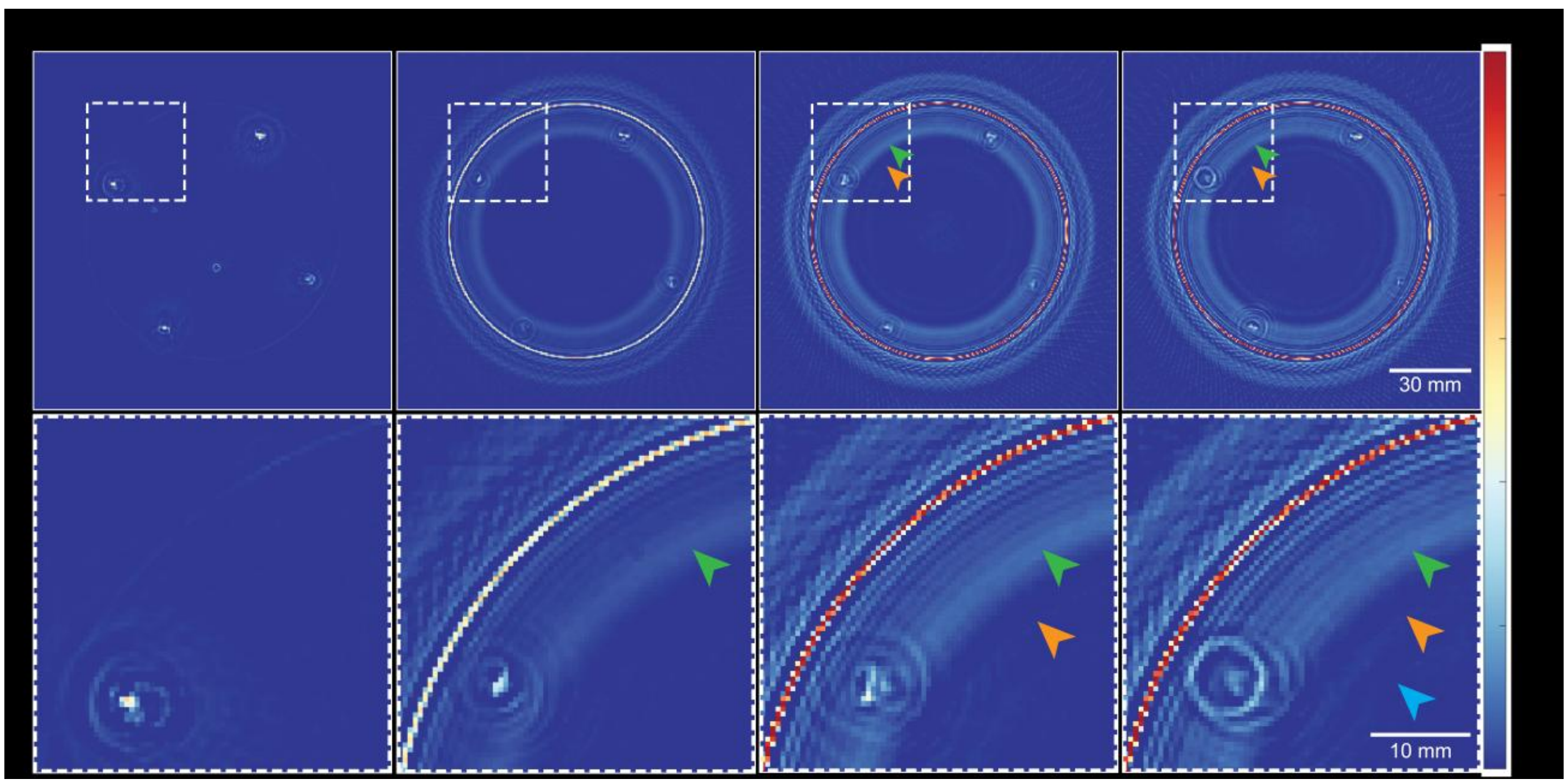

Figure S2 Coronal slices of reconstructed photoacoustic images of four phantoms acquired at 720 nm. **a.** Overview slice with the region of interest indicated by a white box. **b.** Zoomed-in view of the region of interest. Reflection artifacts appear as white rings parallel to the surface; colored arrows denote corresponding artifacts at the same depth across phantoms, with colors indicating identical artifact locations.

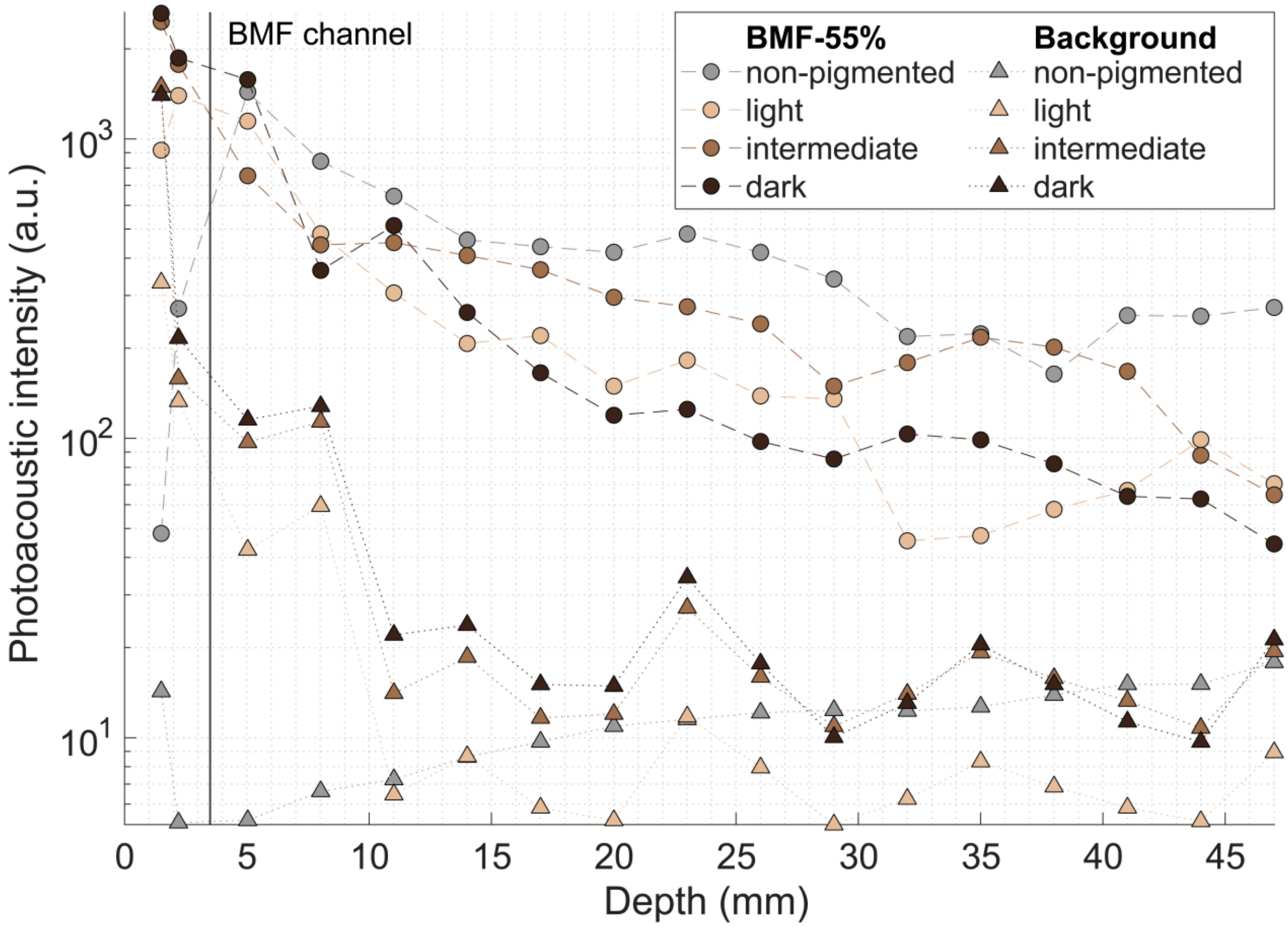

Figure S3 Photoacoustic intensity as a function of depth for The channel filled with s4 at 720 nm for all four phantoms. Dots represent measured signals from the sulfate solution-filled channel, and triangles represent background signals. The channel starts at 3.5 mm below the phantom surface, as indicated by the vertical black line.
